# Clinical and pathological characteristics associated with the presence of the IS6110 *Mycobacterim tuberculosis* transposon in neoplastic cells from non-small cell lung cancer patients

**DOI:** 10.1038/s41598-022-05749-z

**Published:** 2022-02-09

**Authors:** Oscar Arrieta, Camilo Molina-Romero, Fernanda Cornejo-Granados, Brenda Marquina-Castillo, Alejandro Avilés-Salas, Gamaliel López-Leal, Andrés F. Cardona, Alette Ortega-Gómez, Mario Orozco-Morales, Adrián Ochoa-Leyva, Rogelio Hernandez-Pando

**Affiliations:** 1 Thoracic Oncology Unit and Laboratory of Personalized Medicine, Instituto Nacional de Cancerología (INCan), San Fernando #22, Section XVI, Tlalpan, 14080 Mexico City, Mexico; 2 Departamento de Microbiología Molecular, Instituto de Biotecnología, Universidad Nacional Autonoma de México, Cuernavaca, Morelos Mexico; 3 Experimental Pathology Laboratory, Department of Pathology, Instituto Nacional de Ciencias Médicas y Nutrición Salvador Zubirán, Mexico City, Mexico; 4 Department of Pathology, INCan, Mexico City, Mexico; 5 Luis Carlos Sarmiento Angulo Cancer Treatment and Research Center (CTIC), Bogotá, Colombia; 6 Foundation for Clinical and Applied Cancer Research (FICMAC), Bogotá, Colombia; 7 Molecular Oncology and Biology Systems Research Group (FOX-G/ONCOLGroup), Universidad El Bosque, Bogotá, Colombia; 8 Translational Medicine Laboratory, Instituto Nacional de Cancerología (INCan), Mexico City, Mexico

**Keywords:** Cancer, Molecular biology

## Abstract

Lung cancer (LC) and pulmonary tuberculosis (TB) are the deadliest neoplastic and bacterial infectious diseases worldwide, respectively. Clinicians and pathologists have long discussed the co-existence of LC and TB, and several epidemiologic studies have presented evidence indicating that TB could be associated with the development of LC, particularly adenocarcinoma. Nonetheless, this data remains controversial, and the mechanism which could underlie the association remains largely unexplored. Some bioinformatic studies have shown that human cancer biopsies have a very high frequency of bacterial DNA integration; since *Mycobacterium Tuberculosis* (MTb) is an intracellular pathogen, it could play an active role in the cellular transformation. Our group performed an exploratory study in a cohort of 88 LC patients treated at the Instituto Nacional de Cancelorogía (INCan) of Mexico City to evaluate the presence of MTb DNA in LC tissue specimens. For the first time, our results show the presence of the MTb IS6110 transposon in 40.9% (n = 36/88) of patients with lung adenocarcinomas. Additionally, through in-situ PCR we identified the presence of IS6110 in the nuclei of tumor cells. Furthermore, shotgun sequencing from two samples identified traces of MTb genomes present in tumor tissue, suggesting that similar Mtb strains could be infecting both patients.

## Introduction

Two critical diseases that are a frequent cause of morbidity and mortality are tuberculosis (TB) and lung cancer (LC)^1,2^. TB is caused by *Mycobacterium tuberculosis* (MTb) and constitutes the leading cause of death by a single pathogen bacterial agent. Annually, TB causes approximately 1.2 million deaths among HIV negative individuals, according to the World Health Organization (WHO), and incident cases were estimated at 10 million cases (8.9–11.0) in 2019 alone^1^. Moreover, the WHO estimates that approximately 23% of the world population is infected by MTb, though only 1 in 10 individuals will develop active disease^3,4^. The importance of this infectious agent cannot be understated, and although several world regions have gained control through public health measures, other areas are still under a considerable burden by pulmonary TB^1^.

Another relevant disease in terms of global mortality is LC. LC is currently the first cause of cancer-related deaths worldwide, and although some encouraging data emerged in the last years, the survival rate for this neoplasm remains merely at 14–15%^2^. Each year, approximately 1.76 million deaths are attributed to LC, as well as 2.09 million incident cases, most of these diagnosed in the advanced setting, and therapies lack curative intent in most cases^5,6^. Non-small cell lung cancer (NSCLC) accounts for approximately 85% of all LCs, and the most common subtype is lung adenocarcinoma^7^. Although tobacco smoke plays an undeniable role in the development of LC, there is still an essential proportion of patients who develop this disease without a history of smoking^8^. Non-smoker patients comprise approximately 10–20% of lung cancer cases globally, although this number can increase in specific subgroups; for example, among female patients in Mexico, only 30% of cases have a positive smoking history^9^. Several other risk factors have been well-described and causally linked to the development of LC, including radon exposure, asbestos, arsenic, and others^9,10^. Infections also seem to play a role, although their specific mechanism remains elusive. In this regard, a history of TB has been associated with LC development in several epidemiologic studies, particularly for the development of adenocarcinoma^11^.

Animal models have presented experimental evidence regarding the association between TB and LC, in such cases the increased inflammatory process from chronic TB infection induces cell dysplasia and squamous cell carcinoma. Nonetheless, the association in epidemiological studies has been observed in humans between TB and lung adenocarcinoma, rather than squamous cell carcinoma, which would suggest other mechanistic pathways^12^. Interestingly, although the International Agency for Research on Cancer Monographs has identified eleven biological agents as group 1 carcinogens, MTb has not been included in this list^13,14^.

TB and LC co-occurrence have been frequently reported in the literature, though the nature of this observation remains undescribed^11,15^. Although results have been inconclusive, one large cohort in China identified that risk of LC was significantly increased in subjects with a previous TB history^16,17^. Similarly, a prospective cohort in Asia showed an increased incidence of LC in TB patients, a risk which was also increased by presence of other comorbidities including COPD and risk factors such as smoking^18,19^. Interestingly, results also indicate that adenocarcinoma is most frequently associated with a TB history, as reported by a systematic review^11^. Furthermore, Wong et al. found an association between TB and lung adenocarcinoma (OR 1.31, 95% CI 1.03–1.66, p = 0.027) among never-smoking Asian women in a genome-wide association study using Mendelian randomization and pathway analysis^20^. Last, a recent meta-analysis concluded that pre-existing TB increases the risk of LC (RR 2.170 (1.833–2.569). The results emphasize the importance of LC screening in this patient subgroup, as there could be a need for a considerable follow-up after the infection has been treated^21^.

Having a history of TB appears not only to increase the risk of developing LC, but it can also negatively affect prognosis. In a cohort study conducted in the Netherlands which included over 8000 persons and had a follow-up of 18 years a total of 214 cases of LC were found, of which 13 had a history of pulmonary TB. The overall survival of patients with LC and a history of pulmonary TB was significantly lower than patients without a history of TB (HR 2.36, 95% CI 1.1–4.9), with an average result of 311 days difference between the two groups^22^. The role of a previous TB infection in terms of prognosis is scarcely understood, though it might be related to specific molecular alterations and response to treatment. For example, a correlation has been observed between TB and *EGFR* mutations in patients with LC^23^. Further, considerable differences have been observed in response to treatment using tyrosine kinase inhibitors (TKIs)^24^, and could be related to a high expression of Epiregulin in *EGFR* positive tumors^25^. Interestingly, inducible nitric oxide synthase (iNOS) and Epiregulin are highly expressed in chronic inflammatory processes^12,24^.

Previous preclinical and clinical studies have provided information regarding the possible mechanisms underlying the relationship between TB and LC. Mtb induces a strong inflammatory response in the lung tissue of infected hosts, and this can in turn promote cancer development and progression^26–29^. Moreover, the infection is also characterized by the formation of TB-induced scars, a process which has been suggested to play an etiologic role in LC development due to the cellular proliferation which occurs during tissue repair^11,27–30^. The role of angiogenesis has also been explored, which is characteristic during repair processes^31^.

Considering the extensive evidence of LC and TB’s relationship, we sought to explore the presence of MTb genetic material within tissue samples of patients with LC treated at the Instituto Nacional de Cancelorogía (INCan) in Mexico City.

## Results

### Patient characteristics

**Figure 1:**
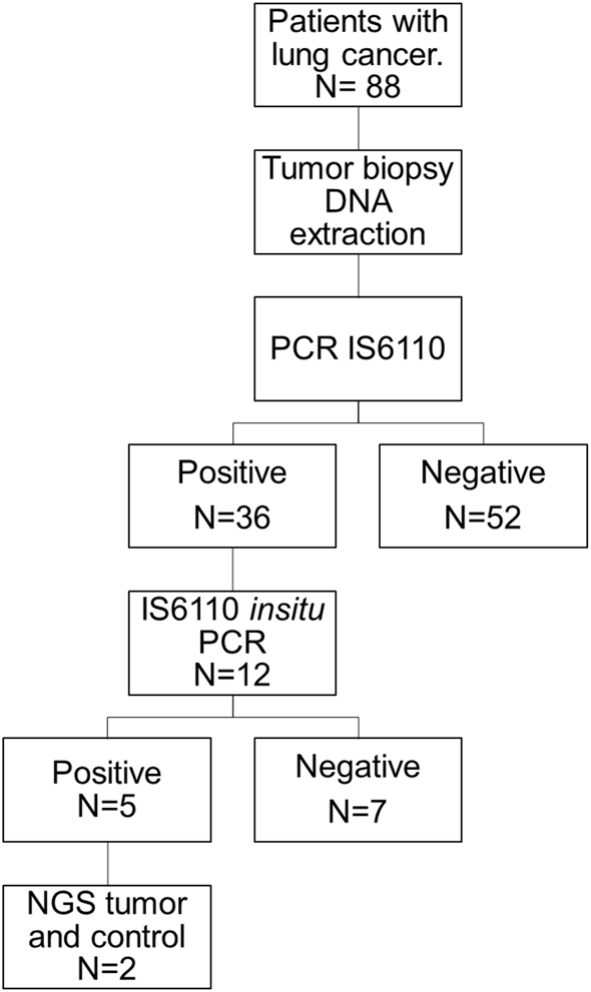
Consort diagram showing the flow of tests and patients included in the study.

The baseline characteristics of the eighty-eight patients included in this study (Fig. 1) are summarized in Table 1. The median age of patients was 60 (21–92) years, and the majority were women 69.3% (61/88). In total, 61.4% (54/88) of the patients did not have tobacco smoke exposure, 56.8% (50/88) had no wood-smoke exposure, 84.1% (74/88) were stage IV at diagnosis, and 93.2% (82/88) had adenocarcinoma. Most patients had an acinar histology subtype (27/88; 30.7%). Only 58% (51/88) of the patients were tested for *EGFR* mutations (*EGFRm)*, and among these 47.1% (n = 24/51) had an *EGFRm.* The most commonly reported *EGFRm* was a deletion in exon 19, present in 54.2% of the cases (13/24) (Table 1).

**Table 1:** Baseline characteristics of NSCLC patients.

Characteristics	Total	IS6110^a^ PCR		*P*-value
	N = 88	Negative n = 52	Positive n = 36	
	% (n/N)	% (n/N)	% (n/N)	
**Sex**				**0.004**
Male	30.7 (27/88)	42.3 (22/52)	13.9 (5/36)	
Female	69.3 (61/88)	57.7 (30/52)	86.1 (31/36)	
**Age at diagnosis (years)**				
Mean (SD)	58.48 ± 15.06	61.06 ± 13.89	54.75 ± 16.07	**0.050**
**Age (years)**				**0.030**
< 60	50 (44/88)	40.4 (21/52)	63.9 (23/36)	
≥ 61	50 (44/88)	59.6 (31/52)	36.1 (13/36)	
**Age category**				0.608
20–39	10.2 (9/88)	7.7 (4/52)	13.9 (5/36)	
40–69	64.8 (57/88)	65.4 (34/52)	63.9 (23/36)	
70+	25 (22/88)	26.9 (14/52)	22.2 (8/36)	
**Body-mass Index (kg/m**^**2**^**)**				
Mean (SD)	25.57 ± 4.18	24.71 ± 4.51	25.96 ± 4.01	0.977
Median (IQR)	24.69 (16.2–34)	24.35 (18.5–32.2)	25.96 (23.1–28.8)	0.916
**Tobacco exposure**				0.968
Absent	61.4 (54/88)	61.5 (32/52)	61.1 (22/36)	
Present	38.6 (34/88)	38.5 (20/52)	38.9 (14/36)	
**Tobacco Index**				
Mean (SD)	28.27 ± 38.31	24.5 ± 31.43	27.5 ± 37.4	0.726
Median (IQR)	20 (1–202)	15 (2–86)	27.5 (1–54)	0.506
**Wood-smoke exposure**				0.811
Absent	56.8 (50/88)	55.8 (29/52)	58.3 (21/36)	
Present	43.2 (38/88)	44.2 (23/52)	41.7 (15/36)	
**Wood-smoke exposure Index**				
Mean (SD)	102.53 ± 156.39	112.16 ± 126.47	15.5 ± 10.6	0.149
Median (IQR)	39 (5–816)	58 (5–300)	15.5 (8–23)	0.731
**Asbestos exposure**				0.837
Absent	90.9 (80/88)	90.4 (47/52)	91.7 (33/36)	
Present	9.1 (8/88)	9.6 (5/52)	8.3 (3/36)	
**Type of disease**				0.692
Loco-regional	40.9 (36/88)	44.2 (23/52)	36.1 (13/36)	
Oligometastatic	55.7 (49/88)	51.9 (27/52)	61.1 (22/36)	
Metastatic	3.4 (3/88)	3.8 (2/52)	2.8 (1/36)	
**Histology**				0.211
Adenocarcinoma	93.2 (82/88)	90.4 (47/52)	97.2 (35/36)	
Other	6.8 (6/88)	9.6 (5/52)	2.8 (1/36)	
**Histology subtype**				0.502
Lepidic	6.8 (6/88)	3.8 (2/52)	11.1 (4/36)	
Acinar	30.7 (27/88)	26.9 (14/52)	36.1 (13/36)	
Papilar	11.4 (10/88)	11.5 (6/52)	11.1 (4/36)	
Solid	26.1 (23/88)	28.8 (15/52)	22.2 (8/36)	
Not specified	25 (22/88)	28.8 (15/52)	19.4 (7/36)	
**Clinical stage**				0.420
I–II	5.7 (5/88)	7.7 (4/52)	2.8 (1/36)	
III	10.2 (9/88)	13.5 (7/52)	5.6 (2/36)	
IV	84.1 (74/88)	78.8 (41/52)	91.7 (33/36)	
**ECOG status**				0.98
0–1	85.2 (75/88)	84.6 (44/52)	86.1 (31/36)	
2 +	14.8 (13/88)	15.4 (8/52)	13.9 (5/36)	
**EGFR mutatio**n				
Wildtype		16	11	0.71
Mutated		13	11	
Not determined (37)		–	–	

Significant values are in bold.

^a^IS6110*: Mycobacterium tuberculosis* insertion sequence.

*NSCLC* Non-small cell lung cancer.

### Patient characteristics according to the presence of the IS6110 transposon detected by End-Point PCR

To evaluate the presence of the IS6110 MTb transposon, End-Point PCR was carried out using DNA isolated from the tumors of 88 patients. Among all samples included, 40.9% (36/88) had a positive End-Point PCR result for the IS6110 transposon. The mean age of positive patients was 54.75 (± 16.07) years. Approximately 63.9% (23/36) of patients positive for IS6110 were younger than 60 years old. In contrast, only 36.1% (13/36) of patients ≥ 61 years old, were positive for IS6110 (*P* = 0.03) (Table 1). Moreover, 86.1% (31/36) of female patients were IS6110 positive, compared with male patients, among who 13.9% (5/36) were positive for the IS6110 transposon (*P* = 0.004). Additionally, 41.7% (15/36) of the patients with brain metastases at diagnosis were positive for IS6110 (*P* = 0.006). Interestingly, the presence of an *EGFR* mutation was not associated with IS6110 (*P* = 0.71) (Table 1).

### Overall survival according to patient characteristics

The median OS for all patients included in the study was 23.1 months (95% CI 7.6–23.2). OS was longer for female patients compared with male patients (20.5 [95% CI 10.9–30.0] vs. 7.4 [95% CI 0.9–13.8]), however the difference did not reach statistical significance (*P* = 0.08). The survival analysis revealed that the only characteristics that were significantly associated with a reduction in OS were histology when comparing adenocarcinoma *vs.* other (17.1 [95% CI 8.6–25.7] vs. 2.1 [95% CI 1.1–3.2]; *P* = 0.001) and adenocarcinoma subtype papillary vs. acinar vs. solid vs. other (25.6 [95% CI 10.6–42.4) vs. 23.0 [95% CI 14.8–31.1] vs. 12.3 [95% CI 2.6–22.0] vs. 4.5 [0.0–10.2]; *P* = 0.02) (Table 2).

**Table 2:** Overall survival according to baseline characteristics.

Characteristic	OS (95% CI)	*P*
**PCR 6110**		
Negative	15.5 (4.1–26.9)	0.58
Positive	15.3 (5.5–25.3)	
**Age**		
< 60	15.5 (5.5–25.5)	0.92
> 60	13.8 (3.6–24.3)	
**Gender**		
Male	7.4 (0.9–13.8)	0.08
Female	20.5 (10.9–30.0)	
**Smoking history**		
No	15.5 (6.5–24.5)	0.31
Yes	15.4 (6.0–24.7)	
**Woodsmoke exposure**		
No	17.1 (3.9–30.3)	0.92
Yes	12.3 (5.3–19.3)	
**Asbestos exposure**		
No	17.1 (8.1–16.2)	0.24
Yes	9.6 (0.0–22.9)	
**ECOG**		
0–1	17.1 (8.7–25.5)	0.06
> 2	7.6 (0.7–14.4)	
**Diabetes**		
No	15.5 (7.1–23.9)	0.97
Yes	13.8 (0.0–30.2)	
**EGFR**		
Wildtype	22.4 (15.0–29.7)	0.93
Mutated	23.0 (8.4–37.5)	
**Histology**		
Adenocarcinoma	17.1 (8.6–25.7)	**0.001**
Other	2.1 (1.1–3.2)	
**Adenocarcinoma subtype**		
Papillary	26.5 (10.6–42.4)	**0.02**
Acinar	23.0 (14.8–31.1)	
Solid	12.3 (2.6–22.0)	
Lepidic	NA	
Not specified/other histology	4.5 (0.0–10.2)	

Significant values are in bold.

### Overall survival according to the presence of the IS6110 transposon

When comparing patients with positive and negative IS6110 PCR results, median OS is 15.3 (95% CI 5.5–25.3) vs. 15.5 (95% CI 4.1–26.9) months (*P* = 0.58). Among patients with a positive IS6110 result, OS was longer for female compared with male patients (17.1 [95% CI 5.6–28.7] vs. 3.8 [95% CI 0.1–7.4]; *P* = 0.28), though this difference did not reach statistical significance. Moreover, patients with adenocarcinoma histology had a prolonged OS compared with other histologic types (15.4 [95% CI 5.6–25.2] vs. 0.9 [95% CI NR]; *P* = 0.001). Interestingly, among patients with a positive IS6110 result, those with an *EGFRm* had a longer OS compared with patients without *EGFRm* (56.0 months [95% CI 14.2–98.0] vs. 21.4 [95% CI 0.0–44.1]; *P* = 0.16), though this did not reach statistical significance*.* No other associations were identified among baseline characteristics for patients with a positive IS6110 (Table 3). Baseline characteristics associated with patients with a negative IS6110 transposon result are summarized in Table 4.

**Table 3:** Overall survival according to baseline characteristics among patients with (+) IS6110.

Characteristic	OS (95% CI)	*P*
**PCR 6110**		
Negative	15.5 (4.1–26.9)	0.58
Positive	15.3 (5.5–25.3)	
**Age**		
< 60	15.5 (5.5–25.5)	0.92
> 60	13.8 (3.6–24.3)	
**Gender**		
Male	7.4 (0.9–13.8)	0.08
Female	20.5 (10.9–30.0)	
**Smoking history**		
No	15.5 (6.5–24.5)	0.31
Yes	15.4 (6.0–24.7)	
**Woodsmoke exposure**		
No	17.1 (3.9–30.3)	0.92
Yes	12.3 (5.3–19.3)	
**Asbestos exposure**		
No	17.1 (8.1-16.2)	0.24
Yes	9.6 (0.0–22.9)	
**ECOG**		
0–1	17.1 (8.7–25.5)	0.06
> 2	7.6 (0.7–14.4)	
**Diabetes**		
No	15.5 (7.1–23.9)	0.97
Yes	13.8 (0.0–30.2)	
**Histology**		
Adenocarcinoma	17.1 (8.6–25.7)	**0.001**
Other	2.1 (1.1–3.2)	
**Adenocarcinoma subtype**		
Papillary	26.5 (10.6–42.4)	**0.02**
Acinar	23.0 (14.8–31.1)	
Solid	12.3 (2.6–22.0)	
Lepidic	NA	
Not specified/other histology	4.5 (0.0–10.2)	

Significant values are in bold.

**Table 4:** Overall survival (OS) according to baseline characteristics in patients with (−) MTb 6110.

Characteristic	OS (95% CI)	*P*
**Age**		
< 60	22.4 (10.3–34.4)	0.57
> 60	21.3 (0.0–62.7)	
**Gender**		
Male	7.4 (0.0–16.5)	0.12
Female	22.4 (9.9–34.8)	
**Smoking history**		
No	15.5 (0.46–30.6)	0.7
Yes	15.4 (0.0–30.8)	
**Woodsmoke exposure**		
No	13.8 (0.0–29.7)	0.61
Yes	15.5 (4.2–26.8)	
**Asbestos exposure**		
No	18 (4.5–31.4)	0.57
Yes	9.6 (2.6–16.7)	
**ECOG**		
0–1	20.5 (10.5–30.5)	**0.01**
> 2	6.8 (0.0–15.7)	
**Diabetes**		
No	15.5 (2.8–28.2)	0.66
Yes	13.8 (0.0–29.7)	
**EGFR**		
Wildtype	26.5 (18.1–34.8)	0.32
Mutated	15.5 (1.6–29.4)	
**Histology**		
Adenocarcinoma	17.9 (5.9–30.0)	**0.01**
Other	2.1 (0.5–1.2)	
**Adenocarcinoma subtype**		
Papillary	22.4 (5.6–39.2)	**< 0.001**
Acinar	25.3 (20–30.6)	
Solid	20.5 (8.8–32.2)	
Lepidic	1.2 (NR)	
Not specified/other histology	4.0 (1.8–6.1)	

Significant values are in bold.

### Presence of the IS6110 transposon detected by in-situ PCR

**Figure 2:**
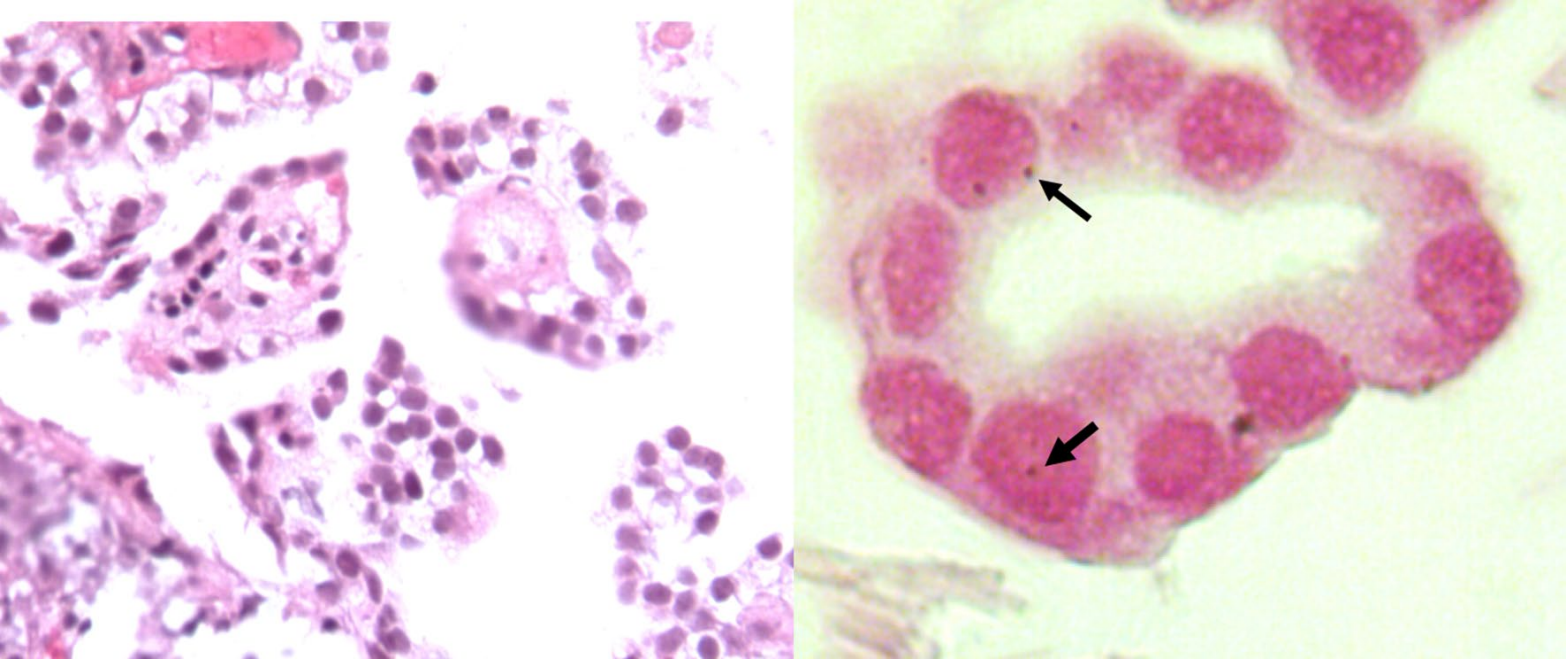
Representative micrographs of pulmonary adenocarcinoma and its in-situ PCR detection of mycobacterial DNA. Left figure shows well differentiated adenocarcinoma stained with haematoxylin/eosin, neoplastic cells show big hyperchromatic basophilic nucleus. The right figure shows high power magnification of neoplastic gland, some cells show positivity for mycobacterial DNA (IS-6110) detected by in situ RT-PCR (dark blue dots, arrows) in the nucleus stained by nuclear fast red.

The samples that resulted positive for IS6110 End-Point PCR were selected for in-situ PCR using the same primers for both experiments. Among 36 tumor samples positive for IS6110,12 had enough paraffin-embedded tumor tissue to perform the *in-situ* PCR test. Among these, 5 were positive (41.6%; n = 5/12), and two of them had the positive signal at the tumor cell nucleus area (Fig. 2). Since the End-Point PCR results for IS6110 suggested the presence of Mtb genome in the tumor, these 2 samples were chosen for tumor sequencing.

### Total shotgun DNA sequencing

**Figure 3:**
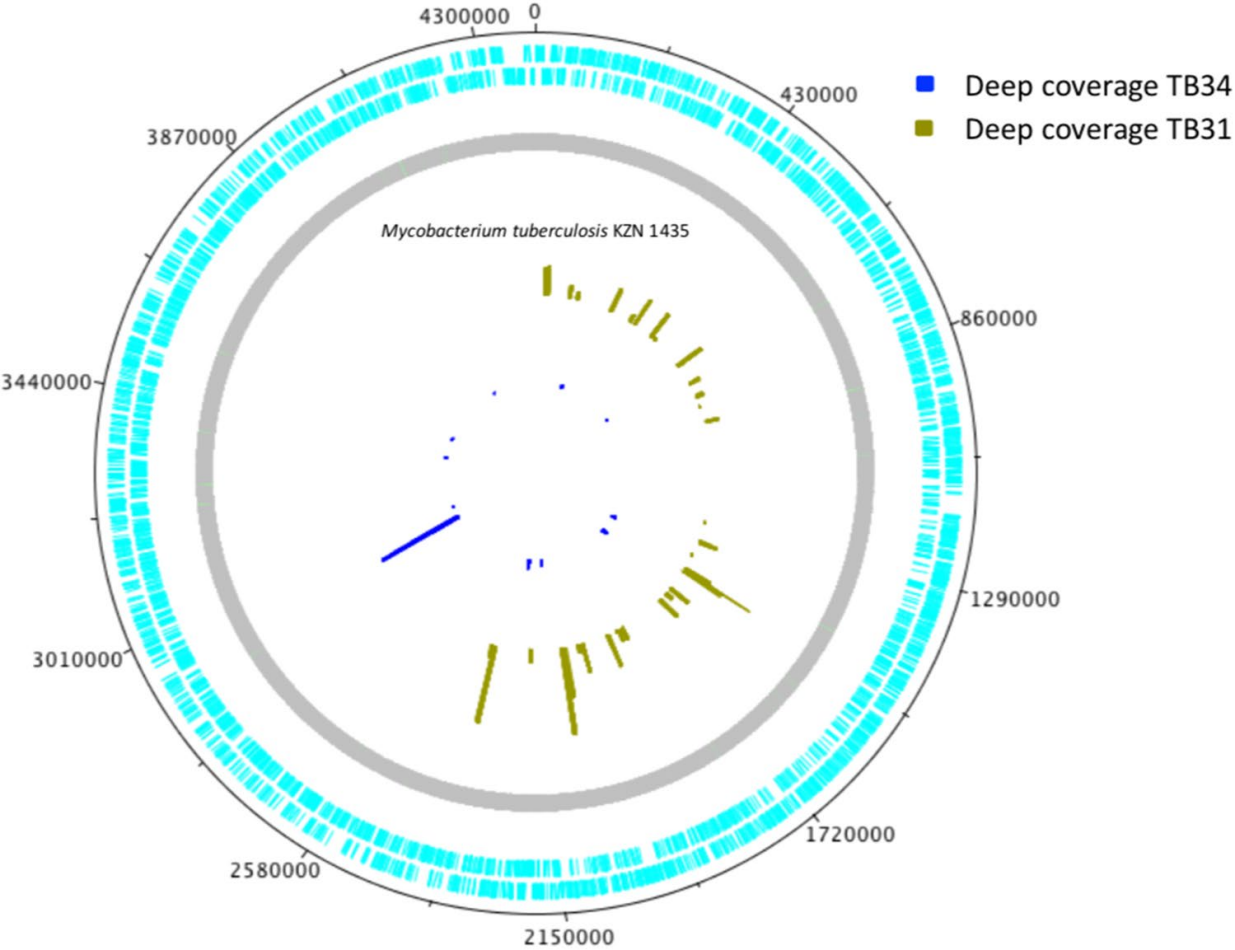
Mapped reads from NGS sequences.

To identify the MTb strain present in the tumor tissues of both sequenced samples (TB31 and TB34), we mapped all sequenced reads against 23 MTb reference genomes (Table 3). After mapping the total reads, we identified 12,191 reads and 10,200 reads that mapped to the reference MTb genomes for TB31 and TB34, respectively. An average of 82.66% of the reads mapped against three strains: *Mycobacterium tuberculosis* KZN 1435 (TB31: 5675 reads and TB34: 4364 reads), *Mycobacterium tuberculosis* str. Haarlem/NITR202 (TB31: 2758 reads and TB34: 3399 reads) and *Mycobacterium tuberculosis* RGTB327 (TB31: 1378 reads and TB34: 891 reads). Interestingly, in both stances reads mapped mainly against MTb strain KZN 1435, suggesting that similar strains could be infecting both patients. It is important to note that the mapped reads were distributed across all the MTb genome (Fig. 3). This result also suggests that the strain present in both patients is closely related to KZN 1435.

## Discussion

Despite the well documented epidemiological association between pulmonary Tb infection and LC, the molecular mechanisms by which Tb promotes the development and progression of LC remain unknown^19^. Nonetheless, the association has been documented even in large population-based studies, highlighting a considerable risk also for secondary lung cancer even after adjusting for important covariates^32^. TB appears to be a significant factor in LC development, though the mechanism behind this association has seldom been explored. It is well known that TB induces an inflammatory response, and that chronic inflammation is conducive to neoplastic processes through several pathways^26–29,31^. Furthermore, other infectious agents have been well characterized for their oncogenic properties, accounting for a considerable proportion of worldwide cancers^33^. Therefore, it is not unreasonable to explore new hypotheses where MTb actively participates in the cellular transformation that leads to cancer. In this study, we evaluated the presence of the IS6110 MTb transposon on the primary tumor DNA of eighty-eight patients through End-Point PCR, and results show that 40.9% (36/88) had a positive End-Point PCR for the IS6110 transposon. The IS6110 transposon is an insertion sequence that is only present in the pathogens of the MTb complex, and therefore the presence of this element in DNA recovered from lung adenocarcinomas, together with the identification of this element through in situ PCR in the nuclei of cancer cells constitutes irrefutable evidence which indicates the presence of the genetic material of MTb in these tumor samples^34^.

This result is higher compared with the reported rate of latent TB infection worldwide (23%)^3^, nevertheless, it must be noted that this cohort of LC patients represents a selected population and therefore a selection bias could be responsible for this difference, compared with open population and other epidemiology reports which have shown an association between LC and TB^26–29,31^. This is not the first time MTb DNA is found in the absence of a TB histological reaction. We previously showed that mycobacterial DNA was identified in 38% and 35% of normal lung tissue samples from Ethiopia and Mexico, respectively, by conventional PCR, in subjects without a previous TB history^35^.

Another interesting observation is the difference in age of patients who tested positive for IS6110 compared with those who tested negative. The mean age of positive patients was 54.75 (± 16.07) years. Among them, 63.9% (23/36) were younger than 60 years old (*P* = 0.03) Our group recently reported that the mean age of patients with LC in Latin America was 62.2 years (± 12.3)^36^ and the median age for this cohort is similar to the one reported for patients with no known risk factors for LC^9^. It is essential to highlight that previous studies have already shown this important association between young age and increased risk of lung cancer among subjects with a TB history. In a study which compared 3776 pulmonary TB patients with 18,880 matched controls, the authors identified that the risk for lung cancer increased as a function of younger age. Compared to patients < 50 years of age, the risks for lung cancer were HR 9.85, 7.1, 3.32, and 2.57 in patients aged 50–59, 60–69, and ≥ 70 years, respectively^37^.

Another critical factor to consider pertaining to the baseline characteristics of patients with a positive IS6110 result is the significant predominance of female sex patients with this characteristics. In a study by Chang et al*.* the authors perform a retrospective cohort among patients with lung cancer receiving *EGFR* targeted therapy, with the objective of assessing whether TB affects the outcome of patients with NSCLC. In this study, the authors identify that a history of TB has a gender-dependent impact^24^. This could be a hypothesis worth exploring, given the association between female sex and *EGFR* mutations, and the possible mechanistic pathway for TB-induced lung adenocarcinoma, which some hypothesize could involve the EGFR pathway. In a study which included 477 patients with pulmonary adenocarcinoma, 39% had *EGFR-*mutated tumors, while 21% had pre-existing TB lesions. In this same study, the authors report that the frequency of *EGFR* mutations is significantly higher in the subgroup of patients with TB lesions, and multivariate analysis revealed that pre-existing TB lesions were an independent factor associated with *EGFR* mutations^38^. Although our study included a small number of *EGFRm* patients due to the fact that many samples were not tested for this alteration, and tissue was not available to perform it at the time. It is important to highlight that among positive IS6110 patients, those with an *EGFRm* had a numerically higher median OS compared with wild-type subjects (56.0 vs. 21.4 months), although the difference is considerable, this did not reach statistical significance, likely due to sample size and patients lost to follow-up. Interestingly, this tendency was not observed for IS6110 negative patients with *EGFR* mutations.

To confirm that MTb was present in the lung cancer tissue without histological evidence of TB lesions, in-situ PCR was performed in samples which had a positive End-Point PCR for the IS6110 transposon. Twelve patients had enough paraffin-embedded tumor tissue to perform in-situ PCR, with a positive result in 41.6% (5/12) of the cases. Among these, 2 samples showed nuclear labeling in neoplastic cells (Fig. 2) and were selected to perform whole-genome sequencing. We found a low number of sequencing reads mapping to MTb genomes and an average of 82.33% were from three strains: *Mycobacterium tuberculosis* KZN 1435, *Mycobacterium tuberculosis* str. Haarlem/NITR202, *Mycobacterium tuberculosis* RGTB327. The reads of both patients were mainly associated with the *Mycobacterium tuberculosis* KZN 1435 strain, suggesting that the MTb strain present in both patients is closely related. Interestingly, MTb KZN 1435 was isolated for the first time from patients in KwaZulu-Natal, South Africa, and is considered as a multiple drug resistant (MDR) strain (resistance to isoniazid and rifampicin)^39^, it belongs to the LAM family of MTb. The observation of bacterial sequences in cancer genomes is not novel; recently, Riley et al. performed a bioinformatic analysis of the nuclear and mitochondrial genome in normal and tumor tissue from publicly available sequence data. They found frequent incidences of LGT involving Acinetobacter- and Pseudomonas-like DNA integrated in the human genomes from samples of acute myeloid leukemia. The cancer samples had a 210-fold higher frequency of integration of bacterial DNA compared with the normal tissue^40^. Although, the recruitment fragments and the End-Point PCR results suggested the presence of the MTb genomes in the tumor, more studies are needed to describe the impact of MTb DNA as a causal role in cancer or is a simple byproduct of carcinogenesis.

The information from this study adds to the body of knowledge which seeks to explore the relationship between TB and LC. Nonetheless, the association is difficult to determine due to several challenges, including the subclinical nature resulting from the primary infection, which makes it difficult to identify when it occurred, and several confounding environmental and host factors which can modulate pathogenesis^33^. Despite the considerable challenges, this is an association worth exploring further due to the considerable impact both in terms of follow-up and screening.

## Conclusion

Results from this study show that in patients with lung adenocarcinoma from Latin America, a large proportion of tumor samples have MTb DNA sequence IS6110, additionally we report that these sequences can be identified in the nuclear area of neoplastic cells by in situ PCR. The shotgun sequencing effort suggests that genotypes of the two sequenced patients could be related. The presence of MTb DNA by PCR in this cohort is significantly associated with sex and younger age. Although chronic inflammation is increasingly implicated as a cancer development mechanism following bacterial infection, proto-oncogene disruption by MTb DNA could provide another mechanism in lung oncogenesis. However, further controlled experiments should be undertaken to assess the possible mechanisms by which TB participates in the development of LC.

## Materials and methods

### Experimental design

The present work was a clinical, longitudinal, prospective, observational and analytical study, using a cohort of lung cancer patients to select a non-probabilistic sample type.

### Patients and tissue samples

From January 2015 to December 2017, patients admitted to the Instituto Nacional de Cancerología (INCan) with a pulmonary lesion suggestive of primary lung carcinoma were biopsied prospectively. Lung tissues were obtained by computer tomography-guided tru-cut (CareFusion, San Diego, CA, USA) in the clinically suspected primary tumor after informed consent was obtained.

Patients with histologic confirmed locally advanced and metastatic lung cancer (stages III B and IV) were eligible for inclusion in the present study. If there was a histologic report that did not indicate primary lung cancer, the patient was excluded. A complete clinical-medical history was included, and all lung tumor specimens were collected after confirmation of diagnosis. This study was conducted according to the principles of the World Medical Association Declaration of Helsinki "Ethical Principles for Medical Research Involving Human Subjects"^41^. All experimental protocols were approved by the Scientific and Bioethical committees at INCan (014/009/ICI, CEI/870/14). All patients provided a signed written consent to participate in genotyping/genomic studies. Primary tumor core biopsy was performed before any treatment, and the specimen was snap-frozen in liquid nitrogen for DNA extraction. A total of 88 tumor samples were included in the study (Consort).

### Statistical analysis

Continuous variables were tabulated as medians with ranges, or as means with standard deviations (SDs), depending on data’s distribution. The distribution was assessed using the Shapiro–Wilk test with a P-value greater than 0.05 considered as normally distributed. Two group comparisons were tested using Student’s t test or Mann–Whitney U depending on data’s distribution. Nominal data was analysed using the chi square (X^2^) test. All data were analyzed using the SPSS package v. 20 (SPSS, Inc., Chicago, Ill, US) following methods previously reported by this research group^42^.

### DNA isolation

DNA was extracted from frozen tumor biopsies, weighted, and cryo-fractured in liquid nitrogen. The procedure for extraction and purification of total DNA from tissue (up to 5 mg tissue) was performed using QIAGEN QIAamp DNA UCP Micro Kit (Cat. 5204).

### End-point and in-situ PCR

End-point PCR evaluated DNA from tumor tissue for the MTb transposon IS6110. The DNA concentration was assessed using ND-1000 Spectrophotometer (NanoDrop Technologies, Wilmington, DE, USA) and the quality by agarose gel. End-Point PCR was performed in a 25 μl mixture containing 100 ng of DNA, HotStar Taq Master Mix (Qiagen) and the following IS6110 primers IS-1 5′CCTGCGAGCGTAGGCGTCGG′3 and IS-2 5′CTCGTCCAGCGCCGCTTCGG′3, as previously described^43^. The PCRs were performed with the following cycling conditions: hold at 95 °C for 15 min; complete 40 cycles of denaturation at 95 °C for 30 s; annealing at 70 °C for 30 s followed by extension at 72 °C for 45 s and a final extension at 72 °C for 10 min. The presence of the transposon product of 123 bp was evaluated in a 2% agarose gel. As a positive control, we used MTb H37Rv DNA^35,44^. The samples that resulted positive for IS6110 End-Point PCR were selected for in-situ PCR with the same primers for IS6110, as previously reported^35^. Briefly, 4 μm sections were obtained using a microtome apparatus from paraffin-embedded tumor tissue and placed on electrostatically charged slides. Incubation removed paraffin at 60 °C for 20 min and then hydrated gradually in xylene, alcohol, and DEPC-water. Tissue permeabilization was performed with chlorhydric acid (0.02 M) for 10 min, then digested with proteinase K (1 mg/Lt) at 37 °C for 30 min and fixed with 20% acetic acid. The reaction mix contained the FastStart Taq DNA polymerase (Roche), dNTPs couples with digoxigenin (PCR DIG labeling Mix, Roche) and the following primers IS-1 5′CCTGCGAGCGTAGGCGTCGG′3 and IS-2 5′CTCGTCCAGCGCCGCTTCGG′3. The in-situ PCR was performed using Amplicover discs and AmpliClips system from Applied Biosystems in a Touchgene thermo cycler, and the cycling conditions were the same as the end-point PCR mention above. The PCR products were detected with an anti-digoxigenin antibody (1:500) incubated for 30 min in a wet chamber; NBT/BCIP (1:50) was used as a substrate incubated for 30 min in a wet chamber and Nuclear Fast Red as counterstaining^35^.

### Shotgun sequencing

Two out of 5 samples that were positive for IS6110 transposon by end-point and in-situ PCRs were subjected to massive sequencing of the total DNA. To this end, the Truseq Nano DNA HT kit (Illumina USA, Cat. No. 20015965) was used to construct each library, starting from 1 μg of tumor genomic DNA, according to the manufacturer’s instructions. The massive sequencing was performed using the Illumina Hiseq 4000 platform at Novogene (CA, USA).

### Bioinformatic analysis

The pair-end reads were quality checked and trimmed (> Q20) using fastx toolkit and trimmomatic software’s. Then, the quality-filtered reads were mapped against 23 complete genomes of *Mycobacterium tuberculosis* strains downloaded from NCBI (Supplementary Table 1) using SMALT (https://sourceforge.net/projects/smalt/). The read count to each genome was obtained using Samtools and Artemis tools, respectively^45,46^. Deep coverage plots were generated using DNAplotter^47^.

## Supplementary Information

Supplementary Table 1.
