# Supplementary material for: Clinical and pathological characteristics associated with the presence of the IS6110 Mycobacterim tuberculosis transposon in neoplastic cells from non-small cell lung cancer patients
Source: Sci Rep. 2022 Feb 9;12:2210. doi: 10.1038/s41598-022-05749-z (PMC8828834; doi:10.1038/s41598-022-05749-z)
Supplement: Supplementary file 1 — Supplementary Table 1. [file 41598_2022_5749_MOESM1_ESM.pdf]

Supplementary Table 1. Mapped reads from NGS sequences.

Accession Number	Genome	Genome Lenght	MappedReads-TB31	MappedReads-TB34
CP001658.1	Mycobacterium tuberculosis KZN 1435	4398250	5675	4364
CP004886.1	Mycobacterium tuberculosis str. Haarlem/NITR202	4404786	2758	3399
CP003233.1	Mycobacterium tuberculosis RGTB327	4380119	1378	891
CP003248.2	Mycobacterium tuberculosis H37Rv	4411709	283	55
AE000516.2	Mycobacterium tuberculosis CDC1551	4403837	244	263
CP005386.1	Mycobacterium tuberculosis CAS/NITR204	4392876	224	209
HE608151.1	Mycobacterium tuberculosis UT205	4418088	205	47
CP002884.1	Mycobacterium tuberculosis CCDC5079	4414325	200	47
CP000611.1	Mycobacterium tuberculosis H37Ra	4419977	195	249
CP001642.1	Mycobacterium tuberculosis CCDC5180	4405981	155	30
AL123456.3	Mycobacterium tuberculosis H37Rv	4411532	120	71
CP005082.1	Mycobacterium tuberculosis str. Beijing/NITR203	4411128	97	51
CP006578.1	Mycobacterium tuberculosis EAI5	4391174	85	69
CP005387.1	Mycobacterium tuberculosis EAI5/NITR206	4390306	83	100
CP001641.1	Mycobacterium tuberculosis CCDC5079	4398812	72	43
CP002992.1	Mycobacterium tuberculosis CTRI-2	4398525	69	40
AP012340.1	Mycobacterium tuberculosis str. Erdman = ATCC 35801	4392353	68	98

CP001976.1	Mycobacterium tuberculosis KZN 605	4399120	66	27
CP001662.1	Mycobacterium tuberculosis KZN 4207	4394985	56	36
CP001664.1	Mycobacterium tuberculosis str. Haarlem	4408224	55	33
CP003234.1	Mycobacterium tuberculosis RGTB423	4406587	42	31
CP000717.1	Mycobacterium tuberculosis F11	4424435	40	21
HE663067.1	Mycobacterium tuberculosis 7199-99	4421197	21	26
